# Immunity to TBEV Related Flaviviruses with Reduced Pathogenicity Protects Mice from Disease but Not from TBEV Entry into the CNS

**DOI:** 10.3390/vaccines9030196

**Published:** 2021-02-26

**Authors:** Monique Petry, Martin Palus, Eva Leitzen, Johanna Gracia Mitterreiter, Bei Huang, Andrea Kröger, Georges M. G. M. Verjans, Wolfgang Baumgärtner, Guus F. Rimmelzwaan, Daniel Růžek, Albert Osterhaus, Chittappen Kandiyil Prajeeth

**Affiliations:** 1 Research Center for Emerging Infections and Zoonoses, University of Veterinary Medicine Hannover, Foundation, Bünteweg 17, 30559 Hannover, Germany; Monique.petry@tiho-hannover.de (M.P.); guus.rimmelzwaan@tiho-hannover.de (G.F.R.); albert.osterhaus@tiho-hannover.de (A.O.); 2 Veterinary Research Institute, Hudcova 70, CZ-62100 Brno, Czech Republic; Palus@paru.cas.cz (M.P.); ruzekd@paru.cas.cz (D.R.); 3 Institute of Parasitology, Biology Centre of Czech Academy of Science, Branisovska 31, CZ-37005 Ceske Budejovice, Czech Republic; 4 Department of Pathology, University of Veterinary Medicine Hannover, Foundation, Bünteweg 17, 30559 Hannover, Germany; Eva.Leitzen@tiho-hannover.de (E.L.); Bei.huang@tiho-hannover.de (B.H.); wolfgang.baumgaertner@tiho-hannover.de (W.B.); 5 Department of Virology, Paul-Ehrlich-Institut, 63225 Langen, Germany; johanna.mitterreiter@pei.de; 6 Innate Immunity and Infection, Helmholtz Centre for Infection Research, 38124 Braunschweig, Germany; andrea.kroeger@med.ovgu.de; 7 Institute of Medical Microbiology and Hospital Hygiene, Otto-von-Guericke University, Leipziger Strasse 44, 39120 Magdeburg, Germany; 8 Center of Behavioral Brain Sciences, Otto-von-Guericke University, 39120 Magdeburg, Germany; 9 Department of Viroscience, Erasmus Medical Center Dr. Molewaterplein 50, 3015GE Rotterdam, The Netherlands; g.verjans@erasmusmc.nl

**Keywords:** tick-borne encephalitis virus, Langat virus, CNS, neuronal damage, virus induced immunity

## Abstract

Tick-borne encephalitis virus (TBEV) is a leading cause of vector-borne viral encephalitis with expanding endemic regions across Europe. In this study we tested in mice the efficacy of preinfection with a closely related low-virulent flavivirus, Langat virus (LGTV strain TP21), or a naturally avirulent TBEV strain (TBEV-280) in providing protection against lethal infection with the highly virulent TBEV strain (referred to as TBEV-Hypr). We show that prior infection with TP21 or TBEV-280 is efficient in protecting mice from lethal TBEV-Hypr challenge. Histopathological analysis of brains from nonimmunized mice revealed neuronal TBEV infection and necrosis. Neuroinflammation, gliosis, and neuronal necrosis was however also observed in some of the TP21 and TBEV-280 preinfected mice although at reduced frequency as compared to the nonimmunized TBEV-Hypr infected mice. qPCR detected the presence of viral RNA in the CNS of both TP21 and TBEV-280 immunized mice after TBEV-Hypr challenge, but significantly reduced compared to mock-immunized mice. Our results indicate that although TBEV-Hypr infection is effectively controlled in the periphery upon immunization with low-virulent LGTV or naturally avirulent TBEV 280, it may still enter the CNS of these animals. These findings contribute to our understanding of causes for vaccine failure in individuals vaccinated with TBE vaccines.

## 1. Introduction

Tick-borne encephalitis virus (TBEV) is an enveloped virus belonging to the family Flaviviridae which contains several other vector-transmitted viruses such as West Nile virus, yellow fever virus, dengue virus, Japanese encephalitis virus, and Zika virus (ZIKV), which all may cause life threatening conditions in humans [1]. TBEV is a major cause of concern in Europe and Asia with currently expanding endemic areas [2]. TBEV is primarily transmitted by a tick-bite of *Ixodes* species, which can lead to strain-dependent outcome of illness [3,4]. The clinical course of infections caused by TBEV strains circulating in Europe often shows a biphasic pattern. Approximately 7–14 days after infection, first flu-like symptoms may appear. After an asymptomatic phase, 20–30% of the patients develop a second phase with headache, high fever, and neurological symptoms as a consequence of severe meningitis and meningioenecephalomyelitis. Of these patients, 2% exhibit long-lasting neurological sequelae [5,6]. Preventive vaccination is the only available specific intervention method for TBE to date. Current TBEV vaccines are based on formalin inactivated whole virus preparations, which have proven to be effective in conferring temporary protection. Therefore repeated vaccinations are needed to induce satisfactory immunity with booster doses being required every 5–10 years [6,7,8,9]. Live attenuated vaccines have been proven to confer better protection, nevertheless associated safety risks have discouraged their usage [10,11]. LGTV, a naturally occurring low-virulent flavivirus, that has high amino acid sequence identity with TBEV (≈80%), has shown potential as a live attenuated vaccine candidate. In Russia LGTV was used in the 1970s to vaccinate against TBE, which was regarded highly successful until among vaccinees displaying neurological symptoms (about 1:20,000) were observed [2,12,13,14,15]. Apparently, this LGTV-based vaccine was under-attenuated. Protection against TBEV and other flaviviruses in the periphery is largely mediated by virus-specific antibodies. In contrast, virus control within the central nervous system (CNS) is dependent on the interplay between infiltrating virus specific T cell and CNS cells. It has been shown that this interaction may have both beneficial and detrimental effects [16,17,18]. Investigations into the pathogenesis of TBE in patients revealed that granzyme B releasing T cells and microglia cells/macrophages contribute to the tissue damage after infection, resulting in neuronal death and astrogliosis [19]. It was recently shown that even low dosage LGTV infection in mice may result in astrogliosis and microglia activation in the hippocampus, suggesting that infection with nonlethal doses of flaviviruses can indeed lead to histopathological changes in the brain [20].

To gain more insights in live vaccine induced protective immunity against TBE, we studied the efficacy of LGTV and naturally avirulent member of the TBEV serogroup (TBEV-280; a strain closely related to another naturally avirulent and well-characterized strain TBEV-263 [21]) in conferring protection against challenge with highly pathogenic TBEV strain in mice. Our findings show the potential of TP21 and TBEV-280 in providing protection against infection with the highly pathogenic TBEV-Hypr strain and advance our understanding of mechanisms within the CNS that may be involved in vaccination breakthroughs.

## 2. Materials and Methods

### 2.1. Mice and Ethics

Six-week old female C57BL/6JOlaHsd (BL6) mice were obtained from Envigo, Inc. (Indianapolis, IN, USA). Mice were housed in isocage systems with individually ventilated cages. Experiments were done in biosafety level 3 laboratories of the Institute of Parasitology, Biology Center of Czech Academy of Sciences, České Budějovice, Czech Republic. The protocol was approved by the Departmental Expert Committee for the Approval of Projects of Experiments on Animals of the Czech Academy of Sciences and the Committee on the Ethics of Animal Experimentation at the Institute of Parasitology (permit No. 29/2016). All experiments were done in accordance with Czech national law guidelines (animal Welfare Act No. 246/1992 Col.) and European Union guidelines for work with animals. 

### 2.2. Viruses

LGTV strain TP21 (referred to as TP21) was isolated 1956 from a pool of hard ticks (*Ixodes granulatus*) of forest rats near Kuala Lumpur, Malaysia [22]. TP21 was grown in Vero B4 and Vero E6 cells and titers were determined by TCID50 assay on Vero E6 cells. Virus was provided the Department of Molecular Immunology, Helmholz Centre for Infection Research, Braunschweig, Germany. Highly virulent TBEV strain TBEV-Hypr (further on described as TBEV-Hypr) was isolated in 1953 from a diseased child in Brno (former Czechoslovakia). The virus was passaged eight times in suckling mice brains before its use in this study. A naturally avirulent strain TBEV-280 was isolated in 1987 from a pool of *I. ricinus* ticks near Kaplice (former Czechoslovakia). The virus was plaque-purified three times, and passaged three times in suckling mice brains, one time in UFK-NB4 cells and one time in SK-N-SH cells before its use in this study. TBEV titers were determined by plaque assay as described in [23]. The TBEV strains were provided by the Collection of Arboviruses, Institute of Parasitology, Biology Centre of the Czech Academy of Sciences, České Budějovice, Czech Republic. 

### 2.3. Immunization Study

Subcutaneous (s.c) administration of 500 pfu of TBEV-Hypr results in 100% lethality in mice. Here, we utilized this mouse model to test the efficacy of TP21 and TBEV-280 in providing protection against lethal TBEV-Hypr challenge. At 28 days prior to TBEV-Hypr challenge, mice were administered s.c with medium (mock), TP21 (10^4^ pfu) or TBEV-280 (10^4^ pfu). Following a s.c challenge with 500 pfu of TBEV-Hypr, development of clinical signs in mice was monitored for a period of 14 days and when clinical score corresponding to humane endpoint was reached, they were sacrificed.

### 2.4. RNA Isolation and Quantitative Real-Time PCR (qPCR)

To determine viral loads in organs, half the brain, spinal cord and spleen were dissected, weighed and homogenized in AVL Buffer (Qiagen, Hilden, Germany). RNA was isolated using QIAamp viral RNA Mini QC Kit (Qiagen) and the QIAcube machine. Viral load was quantified by Taq-Man qPCR using OneStep RT-PCR Kit (Qiagen) and AriaMx Real-time PCR Systems (Agilent, Waldbronn, Germany). TBEV forward primer (5′-3′ GGGCGGTTCTTGTTCTCC), TBEV reverse primer (5′-3′ ACACATCACCTCCTTGTCAGACT), and TBEV probe (5′-3′ TGAGCCACCATCACCCAGACACA) were designed as described in [24]. AriaMX software (version 1.5; Agilent) in combination with intraassay TBEV standard curve was used to analyze the data and viral copies were extrapolated by individual organ weight in RNA copies/gram. qPCR to detect LGTV RNA was performed using LGTV forward primer (5′-3′ AACGGAGCCATAGCCAGTGA), reverse primer (5′-3′AACCCGTCCCGCCA CTC) and probe (5′-3′AGAGACAGATCCCTGATGG) as described in [25]. 

### 2.5. Histology

Brains were cut midsagittal and fixed for 48 h in 4% Histofix (Carl Roth, Karlsruhe, Germany). After fixation organs were stored in PBS till paraffin embedment and cut of serial sections on a microtome (2–3 µm; Leica RM 2035; Leica Instruments GmbH, Nuβloch, Germany). Sections were stained by hematoxylin and eosin (HE) and immunofluorescence (IF).

### 2.6. Immunofluorescence

IF was performed as described before [26]. Briefly, sections of formalin-fixed, paraffin-embedded brains were dewaxed and rehydrated using graded alcohols. For antigen retrieval, sections were heated in a microwave oven (800 W) for 20 min in 0.01 M citrate buffer, followed by application of inactivated goat serum or horse serum, respectively. Afterwards, sections were incubated with primary antibodies (see Table 1) targeting TBEV E-protein (clone 1493) [27], glial fibrillary acidic protein (GFAP, astrocytes) and ionized calcium-binding adapter molecule (Iba-1, microglia/macrophages) overnight at 4 °C. For detecting TBEV-infected astrocytes and microglia, double labeling of TBEV E-protein with GFAP and Iba-1 was performed. Negative control sections were incubated with equally diluted rabbit or mouse serum. For visualization of antigen–antibody reactions, sections were treated with Alexa Fluor 488- or Cy3-labeled secondary antibodies (1:200) for 1 h at room temperature. Nuclei were stained with bisbenzimide (Hoechst 33258, 0.01% in methanol, 1:100; Sigma-Aldrich, Taufkirchen, Germany) and mounted with fluorescence mounting medium (Dako Diagnostika, Hamburg, Germany). Sections were evaluated with a fluorescence microscope (Keyence BZ-9000E, Keyence Deutschland GmbH, Neu-Isenburg, Germany).

### 2.7. Histological Evaluation

HE stained slides were evaluated by semiquantitative scoring of neuronal necrosis, gliosis and inflammation for individual brain regions. The applied scoring system included three categories. Neuronal necrosis scores ranged from 0 to 3 (0 normal; 1 single necrotic neurons, 2 less than 30% affected neurons; 3 more than 30% affected neurons). Gliosis, interpreted as hyperplasia and hypertrophy of glial cells, ranged from 0 to 3 (0 normal; 1 multifocal, mild; 2 multifocal, moderate; 3 multifocal, severe) and inflammation scores ranged from 0 to 3 (0 normal; 1 single perivascular infiltrates; 2 2–3 layers of perivascular infiltrates; 3 more than 3 layers of perivascular infiltrates). TBEV scoring ranged from 0 to 3 (1 <30% positive cells, 2 30–60% positive cells; 3 >60% positive cells). All scorings were performed using a defined area of high power fields (HPFs; 400 x). 

### 2.8. Statistics

Data was analyzed by GraphPad Prism Software 9. Survival curves are displayed by Kaplan–Meier curves using log rank test. Body weights are expressed by mean ± standard error (SD) in graphs and were analyzed using unpaired, two-tailed Student’s t-test. Differences between viral load or HE scorings were analyzed using Mann–Whitney *U* test unless described differently. A *p*-value of < 0.05 was considered significant and indicated by (*).

## 3. Results

### 3.1. TP21 and TBEV-280 Immunization Protects Mice from Lethal TBEV-Hypr Infection

**Figure 1:**
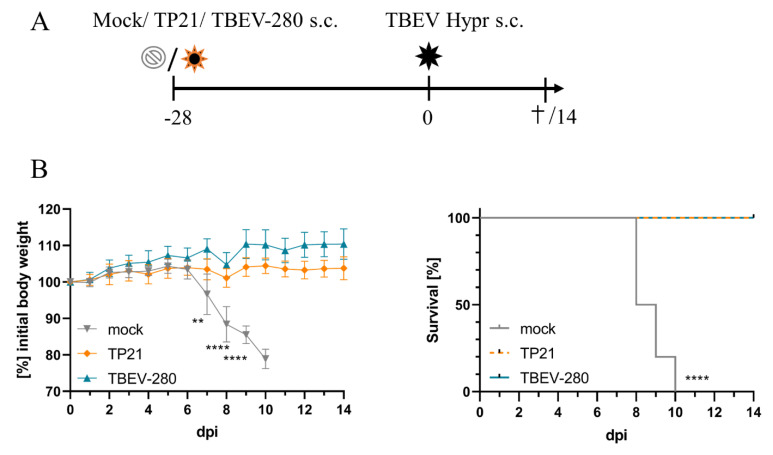
Low-virulent Langat virus (LGTV (TP21)) and naturally avirulent tick-borne encephalitis virus (TBEV (TBEV-280)) immunization protects mice from highly pathogenic TBEV (TBEV-Hypr) challenge († = time of sacrifice). (**A**) Schematic representation of the study. (**B**) Changes in body weight and the survival of the mice post TBEV-Hypr challenge was monitored for a period of 14 days. Data is presented as mean ± SD (*n* = 10) of percentage change relative to initial body weight on the day of TBEV-Hypr challenge. Body weight data was analyzed using unpaired Student’s *t*-test and survival data analyzed by log-rank test (** *p*< 0.01, **** *p*< 0.0001).

First, we tested the efficacy of immunity induced by preimmunization with TP21 or TBEV-280 to confer protection against lethal TBEV-Hypr infection. As demonstrated in Figure 1, all mock-treated mice challenged with TBEV-Hypr started showing clinical signs from 7 dpi onward with body weight loss accompanied by signs of weakness, reduced activity, pilo-erection, kyphosis, and lethargy, often combined with neurological indicators of ataxia and paresis of hind legs. Nearly 50% of mice attained clinical scores corresponding to humane endpoint at 8 dpi and the rest had to be euthanized due to severe disease by 10 dpi. Notably, all mice that were immunized with TP21 or TBEV-280 were protected from developing clinical signs and survived lethal TBEV infection until study endpoint (14 dpi) with no notable changes in the body weights. These results show that preimmunization with TP21 or TBEV-280 is effective in inducing immunity in mice, which conferred protection from clinical and lethal TBEV infection.

### 3.2. Effect of TP21 and TBEV-280 Immunization on TBEV-Hypr Replication in Organs

**Figure 2:**
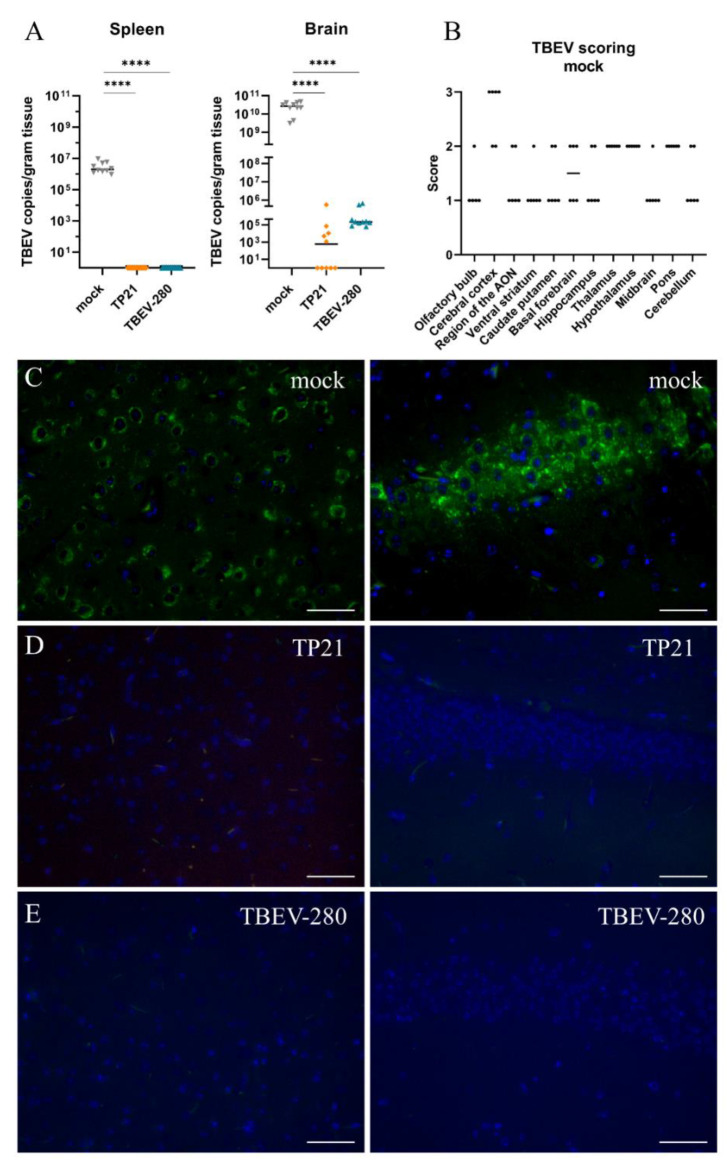
TBEV distribution in the periphery and in the CNS. (**A**) qPCR analysis of viral RNA isolated from the spleen and brain of mock (▼), TP21 (♦) and TBEV-280 (▲) immunized mice. RNA copies per gram tissue were determined and data obtained from individual mice were plotted, bars indicate median (*n* = 10; **** *p* < 0.0001) (**B**). Tissue sections were stained with antibody targeting TBEV E-protein (green) and Hoechst (nuclei, blue) and virus antigen distribution in brain of mock immunized mice was scored based on the frequency of infected cells in the high power fields (HPF) analyzed (*n* = 6). Representative immunofluorescence images from cerebral cortex (left panels) and hippocampus (right panels) regions from mock immunized (**C**), TP21 immunized (**D**), and TBEV-280 immunized (**E**) mice infected with TBEV-Hypr are depicted here. Scale bars 50 µm.

To gain insight into the virus control in immunized mice, we compared distribution of TBEV-Hypr in peripheral organs and in the CNS of the mock-treated, TP21 or TBEV-280 immunized mice after subsequent infection with TBEV-Hypr. As assessed by qPCR analysis designed to detect TBEV RNA, high copy numbers of viral RNA were found in the spleen and brain of mock-immunized mice that were sacrificed between 8 and 10 dpi due to high clinical scores (Figure 2A). Furthermore, neuro-tropism and ability of TBEV-Hypr to predominantly replicate in neural tissue was evident from highest virus load observed in brains as compared to spleens of control mice (Figure 2A). At the study endpoint (14 dpi), viral RNA was not detected in the spleens of any of the mice that experienced a prior immunization with TP21 or TBEV-280, which shows effective protection of peripheral organs from virus replication. In contrast, significant numbers of viral RNA copies were detected in the brains of some of the TP21 and all of the TBEV-280 preimmunized mice (Figure 2A). However, viral copy numbers detected in these mice were significantly lower than those of mock control mice. Similar observations were made by qPCR analysis of the spinal cords (Supplementary Figure S1). TBEV primers used in this assay do not detect LGTV RNA at numbers less than 10^5^ copies (Supplementary Figure S2). Since viral copy numbers detected in LGTV immunized mice are below detection limit we can confirm that only TBEV RNA is detected in these mice. Immunostaining of brain sections for TBEV antigen confirmed presence of TBEV-Hypr infected cells across different regions of brains of mock control mice. Cerebral cortex, thalamus, hypothalamus, and pons appeared to be the regions with highest viral loads (Figure 2B,C). Interestingly, no TBEV antigen was detected in TP21 or TBEV-280 immunized mouse brains (Figure 2D,E). Taken together these results confirm that TP21 and TBEV-280 immunization qPCR positive induces protective immune response against TBEV-Hypr and effectively keeps virus replication and spread under control. Apparently, virus escaping from peripheral immunity may enter the CNS and replicate there before being cleared by local inflammatory or immune mechanisms. 

### 3.3. TBEV Infects Neurons

**Figure 3:**
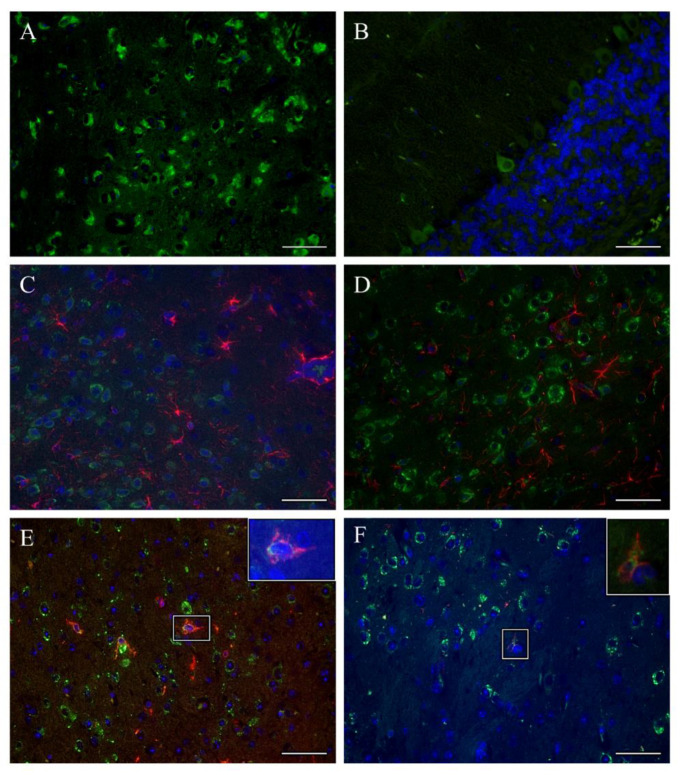
Immunofluorescence determined the cellular targets of TBEV infection in brain. Brain slices were stained using antibody targeting TBEV E-protein (green) and Hoechst (nuclei, blue). Representative images from thalamus (**A**) and cerebellum (**B**) show infected cells that morphologically resemble neurons. Double labeling of TBEV E-protein (green) and glial fibrillary acid protein (GFAP, astrocytes; red) of hypothalamus (**C**) and cerebral cortex (**D**) shows no colocalization of TBEV antigen with astrocytes. Costainings with anti-Iba-1 (microglia/macrophages marker; red) and TBEV E-protein (green) show mostly uninfected microglia/macrophages. However, few Iba-1^+^ TBEV^+^ cells were detected in the midbrain (**E**) and thalamus (**F**) of mock immunized and TBEV-Hypr infected mice. Scale bars 50 µm.

Immunofluorescence staining for TBEV antigen of brain sections from control, TBEV-Hypr-infected mice revealed high numbers of infected cells across different brain regions. Based on location and morphology, most of the infected cells appeared to be neurons (Figure 3A,B), as has been reported previously [28,29]. However, to further investigate whether major glial cell types such as microglia and astrocytes could also constitute a target of TBEV, double labeling of brain sections using antibodies targeting TBEV antigen and microglia/macrophages (Iba-1^+^) or astrocytes (GFAP^+^) was performed. Interestingly, none of the regions that were screened displayed astrocytes (GFAP^+^ cells) that colocalized with TBEV antigen (Figure 3C,D). Largely, microglia/macrophages (Iba-1^+^ cells) also failed to show colocalized TBEV antigen. However, in some areas of cerebral cortex and midbrain of single TBEV-Hypr infected control mice we detected Iba-1^+^ cells costained with TBEV antigen (Figure 3E,F). Collectively these results confirm that neurons constitute the primary target of TBEV infection. 

### 3.4. Marked Neuronal Necrosis Observed after TBEV-Hypr Infection in Mock Control Mice

**Figure 4:**
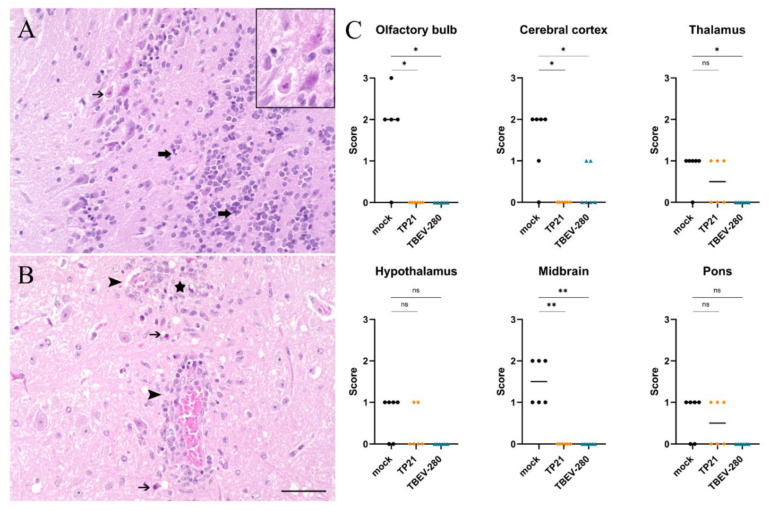
Assessment of neuronal damage in brain following TBEV-Hypr challenge. Representative images of HE staining from olfactory bulb (**A**) and thalamus (**B**) of mock immunized and TBEV-Hypr infected mice demonstrating neuronal necrosis (thin arrows), inflammation (arrow heads), cellular debris/pyknotic nuclei (thick arrows), and gliosis (star) are shown here; scale bars 50 µm. (**C**) Neuronal necrosis in different regions of brain of mock (●), TP21 (♦) and TBEV-280 (▲) immunized and TBEV-Hypr infected mice (*n* = 6) were scored on a scale of 0–3 (see methods). Each dot represent individual mice, bars indicating mean value (* *p* < 0.05, ** *p* < 0.01, ns = not significant).

The results so far indicate that TBEV infects and replicates within neurons upon entering the CNS. Histopathological analysis of HE stained brain sections revealed significant neuronal necrosis in mock immunized mice that were challenged with TBEV-Hypr (Figure 4A,B). The extent of neuronal damage was assessed on a scale of 3 based on the presence of single scattered necrotic neurons per HPF (score 1) or greater than 30% affected neurons per HPF (score 3) in the regions evaluated. Neuronal damage was more prominent in the olfactory bulb, cerebral cortex and midbrain regions of heavily infected brains of mock treated mice. Interestingly, neuronal necrosis was also detected in thalamus, hypothalamus and pons of some of the TP21 immunized mice and occasionally in the cerebral cortex of TBEV-280 immunized mice (Figure 4C and Supplementary Figure S3). This indicates that the TBEV specific immune response induced by TP21 and TBEV-280 immunization protects mice from clinical signs but does not fully protect all immunized mice from neuronal damage.

### 3.5. Inflammation and Gliosis in the Brain

**Figure 5:**
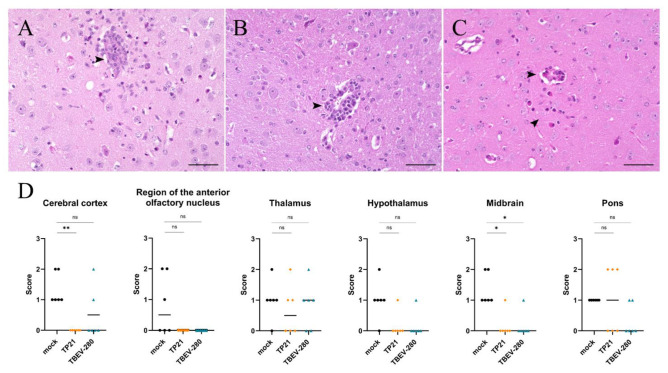
Inflammation in the brain. Regions with perivascular infiltrates (PVI) were assessed using HE staining. PVI (arrow heads) were not only detected in mock (**A**) but also in TP21 (**B**) and TBEV-280 (**C**) immunized mice that were subjected to TBEV-Hypr challenge; scale bars 50 µm. (**D**) High power fields from different regions of brain of mock (●), TP21 (♦) and TBEV-280 (▲) immunized mice (*n* = 6) were screened and the score (see methods) obtained for PVI from individual mice was plotted here. Dots representing individual animals, bars indicating mean value (* *p* < 0.05; ** *p* < 0.01, ns = not significant).

**Figure 6:**
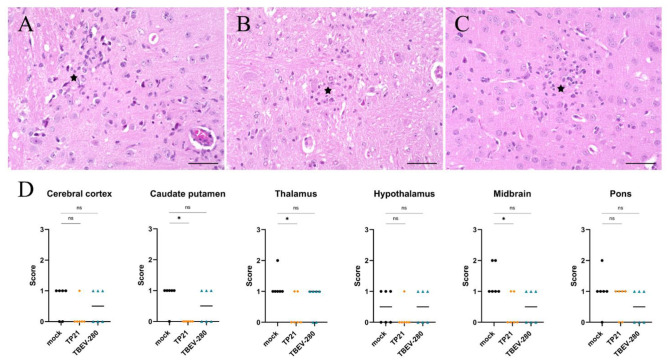
Reactive changes in the brain following TBEV-Hypr challenge. Gliosis (star) was detected in the brain of mock (**A**), TP21 (**B**) and TBEV-280 (**C**) immunized mice that were subjected to TBEV-Hypr challenge; scale bars 50 µm. (**D**) Gliosis was scored on a scale of 0–3 (see methods) by screening HPF from different brain regions of mock (●), TP21 (♦) and TBEV-280 (▲) immunized mice. Dots represent individual animals, bars indicates mean value (*n* = 6; * *p* < 0.05, ns = not significant).

Neuroinfection often results in extensive inflammation within the CNS which is usually characterized by perivascular infiltrates (PVI) of recruited peripheral inflammatory cells as well as hypertrophy and hyperplasia of local glial cells (gliosis). Such changes are not observed in brain of naïve mice. Examination of HE stained brain sections revealed significantly more PVI of mononuclear cells across different regions of brains of mock- immunized mice compared to TP21- and TBEV-280-immunized mice (Figure 5 and Supplementary Figure S4). Similarly, areas of gliosis were more prominent in mock immunized mice than in TP21 and TBEV-280 immunized mice (Figure 6 and Supplementary Figure S5), in line with the attenuated course of disease after immunization. Interestingly, inflammatory infiltrates as well as multifocal, mild accumulation of glial cells were also detected within the brains of TP21 and TBEV-280 immunized mice. This again shows that TBEV possibly gained access to the CNS despite the presence of peripheral protective immunity and triggers neuroinflammation.

## 4. Discussion

In the present study, we showed that immunization of mice with LGTV, a closely related low-virulent flavivirus, or a naturally avirulent TBEV strain (TBEV-280) provided protective immunity in mice against symptomatic and lethal infection with TBEV-Hypr strain of TBEV. Interestingly, traces of viral RNA and signs of neuroinflammation were found in the brains of LGTV and TBEV-280 immunized mice that did not display any clinical signs upon lethal TBEV-Hypr challenge. This is of particular interest in light of breakthrough TBE that have been reported in vaccinated individuals [30,31]. 

Neuronal cells are regarded as primary targets of TBEV [28,29]. Accordingly, in the absence of vaccine-induced protective immunity, high numbers of infected neurons were detected by immunostaining in TBEV-Hypr infected mice. As we could not detect viral antigen within neurons of immunized mice, our initial assumption was that immune response induced by LGTV and TBEV-280 preimmunization effectively cleared TBEV-Hypr from the periphery and prevented its spread into the CNS. However, detailed qPCR analyses of brain and spinal cord from these mice reflected a different outcome. Indeed, the average viral RNA copy number detected in TP21 or TBEV-280 immunized mice was several folds lower when compared to the numbers detected in mock immunized mice. The observed viral RNA copy numbers by qPCR in the brains of the TP21 or TBEV-280 immunized mice (to the order of 10^5^ genome copies/g) were significantly higher than could be expected without active virus TBEV replication. Hence, the most likely explanation for the presence of high viral RNA copy numbers in brains and spinal cords of the immunized mice is that TBEV-Hypr is able to enter the CNS of immunized mice and probably replicate within the neural tissue. This hypothesis is furthermore supported by the presence of inflammatory (PVI) and reactive (gliosis) changes within the CNS of vaccinated mice, which both constitute frequent findings during and after neuroinfection [18]. Besides neurons, some studies have demonstrated TBEV infection of astrocytes [28,32,33]. Furthermore, it has been shown that TBEV can infect and survive in rat and human astrocytes for several days without affecting each other’s viability [32,33]. However, costaining of brain tissue sections did not provide evidence of TBEV antigen within astrocytes either in mock-, LGTV- or TBEV-280-immunized mice after challenge. Similarly, only isolated microglia cells in certain brain regions in mock immunized mice showed colabeling of Iba-1 with TBEV antigen. Microglia are highly efficient phagocytes and actively scavenge cell debris in the event of tissue damage. Therefore, it remains unclear whether detected signal resulted from infection of Iba1-positive cells or from phagocytosis. There is evidence that neurotropic flaviviruses can influence the behavior of microglia and astrocytes and either assist in viral clearance or augment neuropathogenesis by producing toxic neuroinflammatory mediators [17,20,34,35,36]. To avoid any permanent damage, inflammation within the CNS is highly regulated and is primarily mediated by microglia and astrocytes. In response to any perturbation caused within the CNS, these glial cells proliferate and migrate to the affected areas and provide neuroinflammatory mediators and neurotrophic factors to limit the damage [37,38,39]. This phenomenon coined reactive gliosis [40] is characteristic of neural tissue damage caused by injury or infection, which could be also have occurred within the present study. In mock immunized mice after TBEV-Hypr challenge, we also observed gliosis, particularly in those areas with increased viral load. Interestingly, mild areas of gliosis have also been observed in certain brain regions of the TP21 and TBEV-280 immunized mice after TBEV-Hypr challenge, indicating reactive changes within CNS even after immunization. Since we could not detect TBEV antigen in these brains it is difficult to conclude that the areas with gliosis directly correspond to those with assumed previous virus infection. Taken together these findings are highly indicative of TBEV gaining access to the CNS and triggering inflammation despite of pre-existing TP21 or TBEV-280 induced immunity. We observed the presence of PVI in cerebral cortex, thalamus, hypothalamus, and midbrain regions of mock immunized mice. In contrast, PVIs were limited to certain brain regions in immunized mice. This again may reflect differences in virus distribution within the CNS of mock immunized and immunized mice. 

Extensive neuronal necrosis was found in infected brains of mock immunized mice. There was severe loss of neurons especially in the olfactory bulb, cerebral cortex and midbrain regions accompanied by detection of numerous neurons staining positive for TBEV antigen by IF as well as inflammatory and reactive changes. However, it is not clear if the observed neuronal damage represents a direct effect of virus infection or a secondary consequence of inflammation. It has been shown for other flaviviruses that infection and virus replication can trigger apoptosis and necrosis in neurons [19,29,41,42,43,44,45]. Similarly, inflammation as a consequence of neuroinfection can also cause substantial neuronal loss [46,47]. 

Probably the most intriguing finding from this study is the detection of neuronal necrosis combined with inflammation and gliosis despite of vaccine-induced protective immunity to TBEV in some mice. This phenomenon was more prominent in TP21 than in TBEV-280 immunized mice. This is of special interest, considering breakthrough TBE in vaccinated individuals. The observation that TBEV may still enter the CNS despite vaccine induced protective immunity while causing inflammation and neuronal damage may at least in part explain vaccination breakthroughs in TBE vaccinated individuals. 

## 5. Conlusions

In conclusion, this study demonstrates for the first time the presence of TBEV in the CNS of immunized mice that are protected from lethal or clinically manifested infection. Currently, it is unclear if the protection is mediated by specific antibodies, T cells, or a combination of both. Nevertheless, it has been demonstrated for other closely related flaviviruses that specific CD4^+^ and CD8^+^ T cells induced against one virus (e.g., ZIKV) can also provide cross-protection against another related flavivirus (e.g., DENV) [48,49,50]. However, absence of detectable TBEV antigen in these brains after immunofluorescence staining also hints at an important role of local inflammatory mediators in checking the virus spread within the CNS in the event virus escapes host immune response in the periphery. Nonetheless, the actual cause of the neuronal necrosis in immunized mice remains unclear. Whether this is caused by virus infection directly or a consequence of subsequent inflammation or even the action of infiltrating virus-specific T cells remains a matter of further investigation. Recently Garber et al. demonstrated that ZIKV specific T cells through microglia mediate neuronal loss and hence result in cognitive dysfunction [17]. Furthermore, CD8^+^ T cells that clear infected cells also cause neuronal damage [18,19,51]. Finally, it is tempting to speculate whether subtle neuronal necrosis as observed in immunized mice is the reflection of a broader challenge of raising sufficient immune mediated protection of the CNS from TBEV invasion, which may also be the basis of current human vaccination failures.

## Figures and Tables

**Table 1:** Antibodies and reagents of immunofluorescence.

Staining	1st Antibody			Pretreatment	Blocking Serum	2nd Antibody
	Target	Company	Dilution			
Single staining	TBEV E protein	Clone 1493 Andreas Niedrig	1:250	Citrate buffer	Horse	Donkey-anti-mouse alexa flour 488 (Dianova 715-546-150)
Double staining	GFAP	Dako Z0334	1:400	Citrate buffer	Goat	Goat-anti-rabbit-Cy3 (Dianova 111-165-144)
	TBEV E protein	Clone 1493 Andreas Niedrig	1:250	Citrate buffer	Goat	Goat-anti-mouse alexa fluor 488 (Dianova 115-545-003)
Double staining	Iba-1	FUJIFILM 011-27991	1:400	Citrate buffer	Horse	Donkey-anti-goat-Cy3 (Dianova 705-165-147)
	TBEV E protein	Clone 1493 Andreas Niedrig	1:250	Citrate buffer	Horse	Donkey-anti-mouse alexa fluor 488 (Dianova 715-546-150)

## Data Availability

Data is contained within the article or Supplementary Material.

## Supplementary Materials

The following are available online at https://www.mdpi.com/2076-393X/9/3/196/s1, Figure S1: Taq-Man qPCR analysis of the spinal cord of mock, TP21 and TBEV-280 immunized mice that were infected with TBEV-Hypr; Figure S2. (**A**) Sequence alignment of TBEV Hypr (Genbank KP716974.1), LGTV TP21 (Genbank EU790644.1) and TBEV-280 (sequence unpublished) highlighting the attachment of primers and probe to detect TBEV template in mouse samples (Geneious Prime software). (**B**) Geneious Prime software was used to determine the sequence identity between all three viruses. (**C**) A comparison of a qPCR analysis using TBEV and LGTV primers of LGTV RNA (isolated from TP-21 stock) and RNA isolated from brain of LGTV- and TBEV-280-immunized mice that were challenged with Hypr. qPCR results were analyzed by AriaMx Software (Agilent) displayed in mean values (of duplicates); Figure S3: Evaluation of neuronal necrosis by HE staining in other brain regions. Regions were scored from 0 to 3 (see methods) and statistical analyzed by Mann-Whitney test (* *p* < 0.05; *n* = 6/group); Figure S4: Evaluation of inflammation using HE staining in different brain regions. Regions were scored from 0 to 3 (see methods) and statistical analyzed by Mann-Whitney test (* *p* < 0.05; *n* = 6/group); Figure S5: Evaluation of gliosis using HE staining in different brain regions. Regions were scored from 0 to 3 (see methods) and statistical analyzed by Mann-Whitney test (* *p* < 0.05; *n* = 6/group).
