# Supplementary material for: Immunity to TBEV Related Flaviviruses with Reduced Pathogenicity Protects Mice from Disease but Not from TBEV Entry into the CNS
Source: Vaccines (Basel). 2021 Feb 26;9(3):196. doi: 10.3390/vaccines9030196 (PMC7996866; doi:10.3390/vaccines9030196)

Supplementary Figure S1.

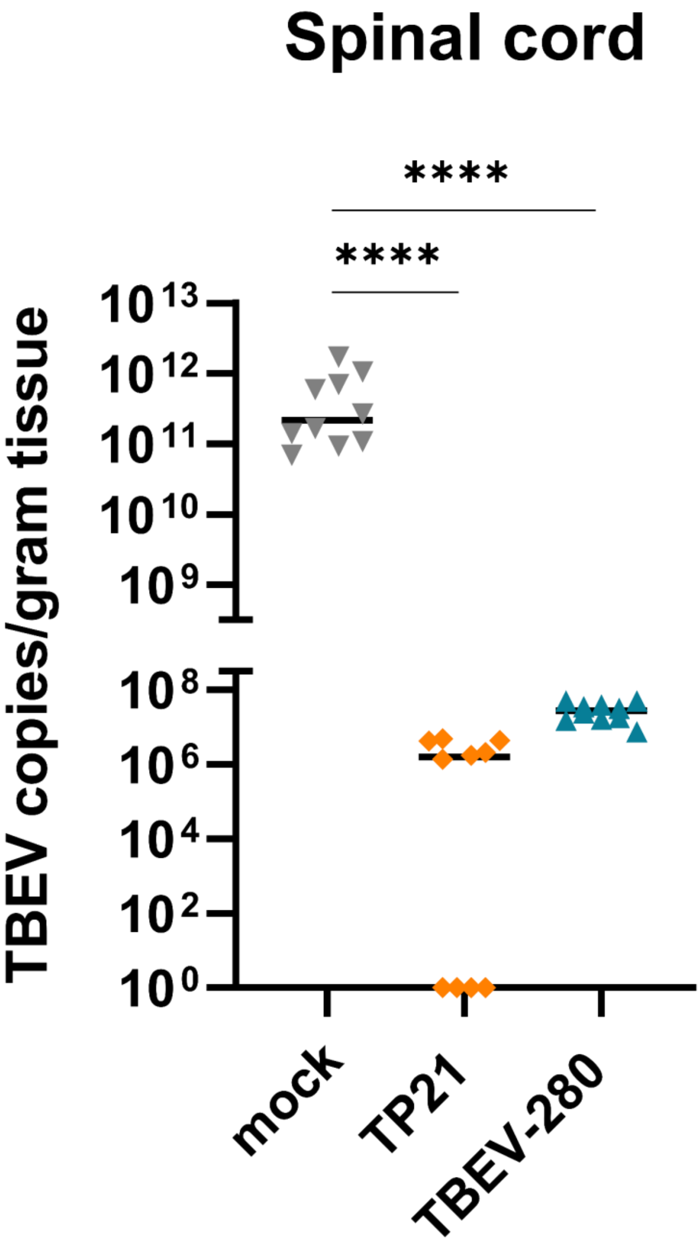

Supplementary Figure S2.

A

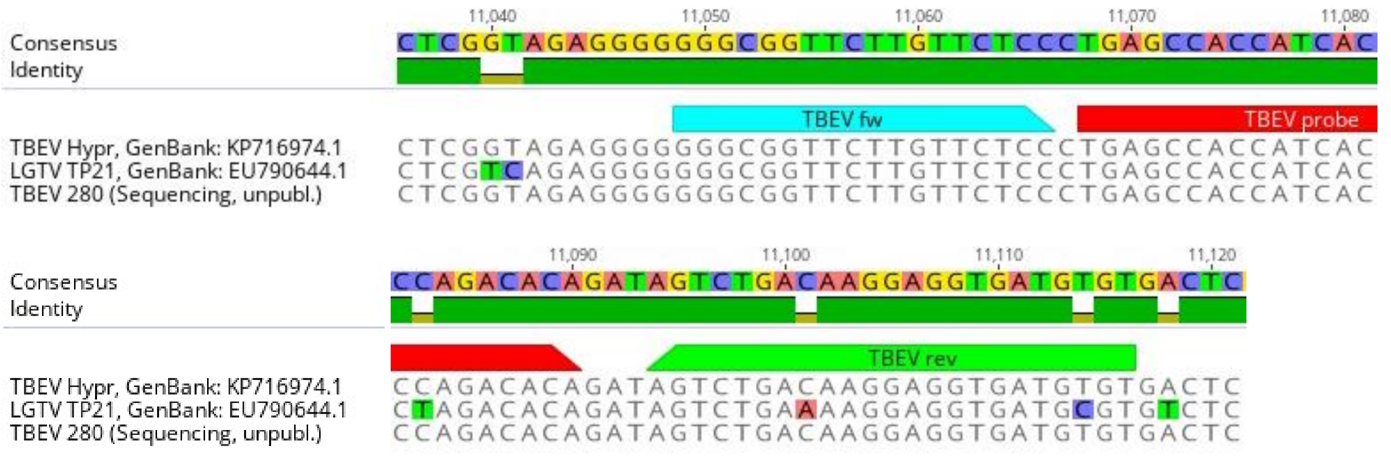

B

	**LGTV TP21 (Genbank EU790644.1)**	**TBEV 280 (Sequencing, unpubl.)**
**TBEV Hypr (Genbank KP716974.1)**	73.84 %	97.45 %

C

	**TBEV primer**		**LGTV primer**	
	**Ct value**	**total copy no./ reaction**	**Ct value**	**total copy no./ reaction**
LGTV TP21 stock 1:10	29,95	1,89E+04	19,77	1,72E+09
LGTV TP21 stock 1:100	32,37	4,09E+03	22,92	1,38E+08
LGTV TP21 stock 1:1000	36,44	3,10E+02	26,74	6,47E+06
LGTV TP21 stock 1:10000	No Cq	No Cq	30,12	4,30E+05
Mouse brain sample LGTV TP21 + TBEV-Hypr inf	No Cq	No Cq	No Cq	No Cq
Mouse brain sample LGTV TP21 + TBEV-Hypr inf	No Cq	No Cq	33,51	2,92E+04
Mouse brain sample LGTV TP21 + TBEV-Hypr inf	34,97	8,92E+02	33,68	2,53E+04
Mouse brain sample TBEV-280 + TBEV-Hypr inf	35,7	5,62E+02	No Cq	No Cq
Mouse brain sample TBEV-280 + TBEV-Hypr inf	34,08	1,57E+03	No Cq	No Cq

Supplementary Figure S3.

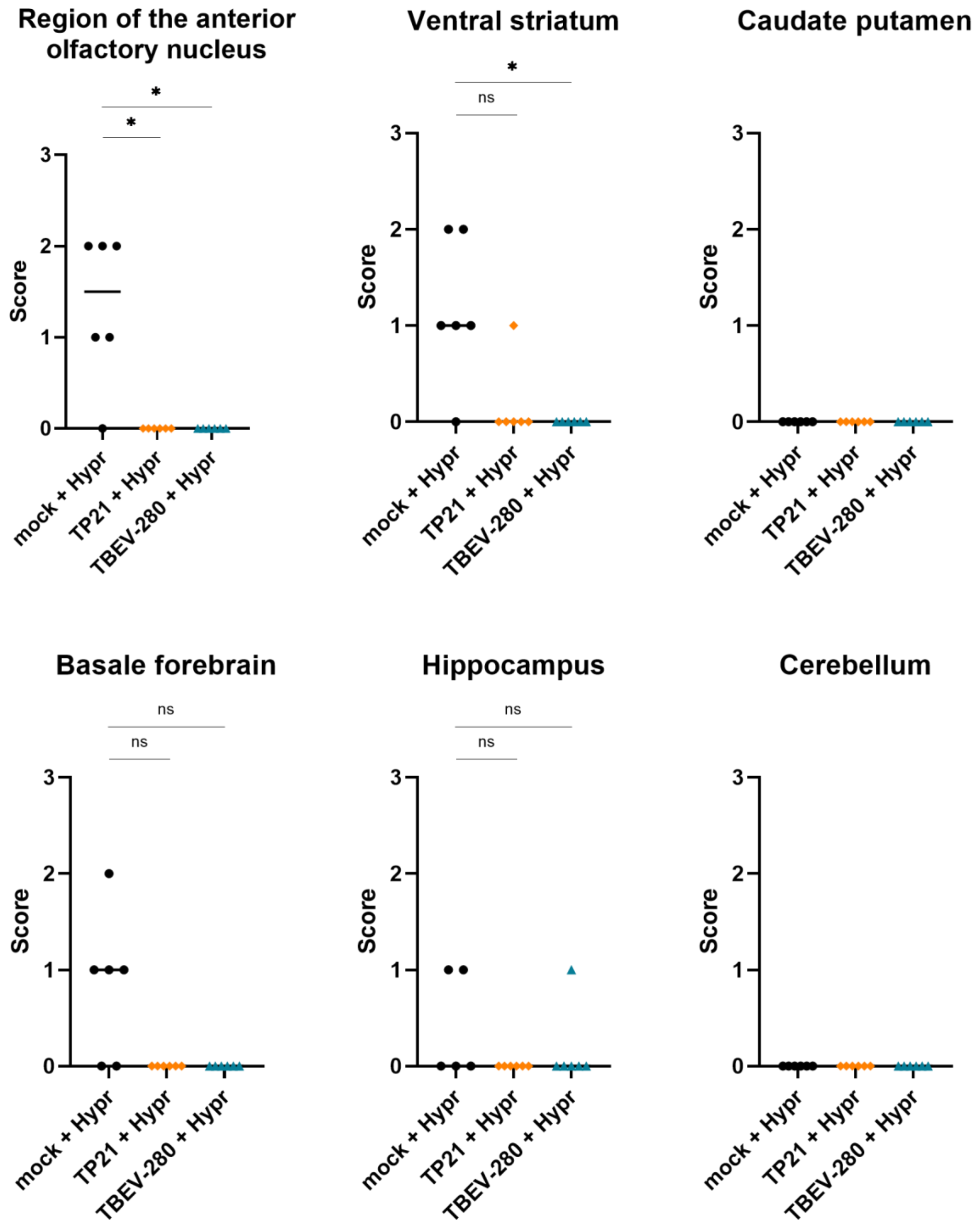

Supplementary Figure S4.

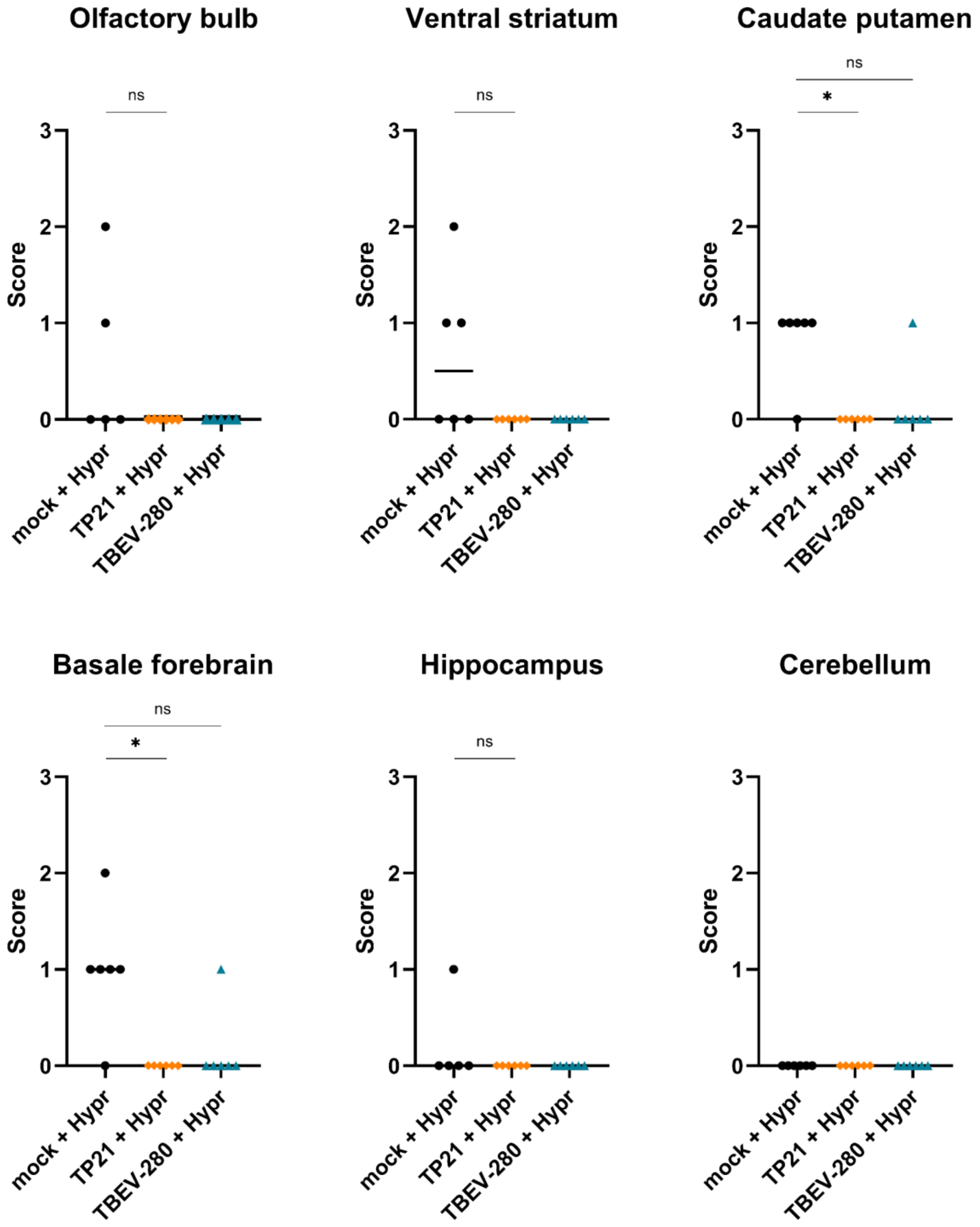

Supplementary Figure S5.

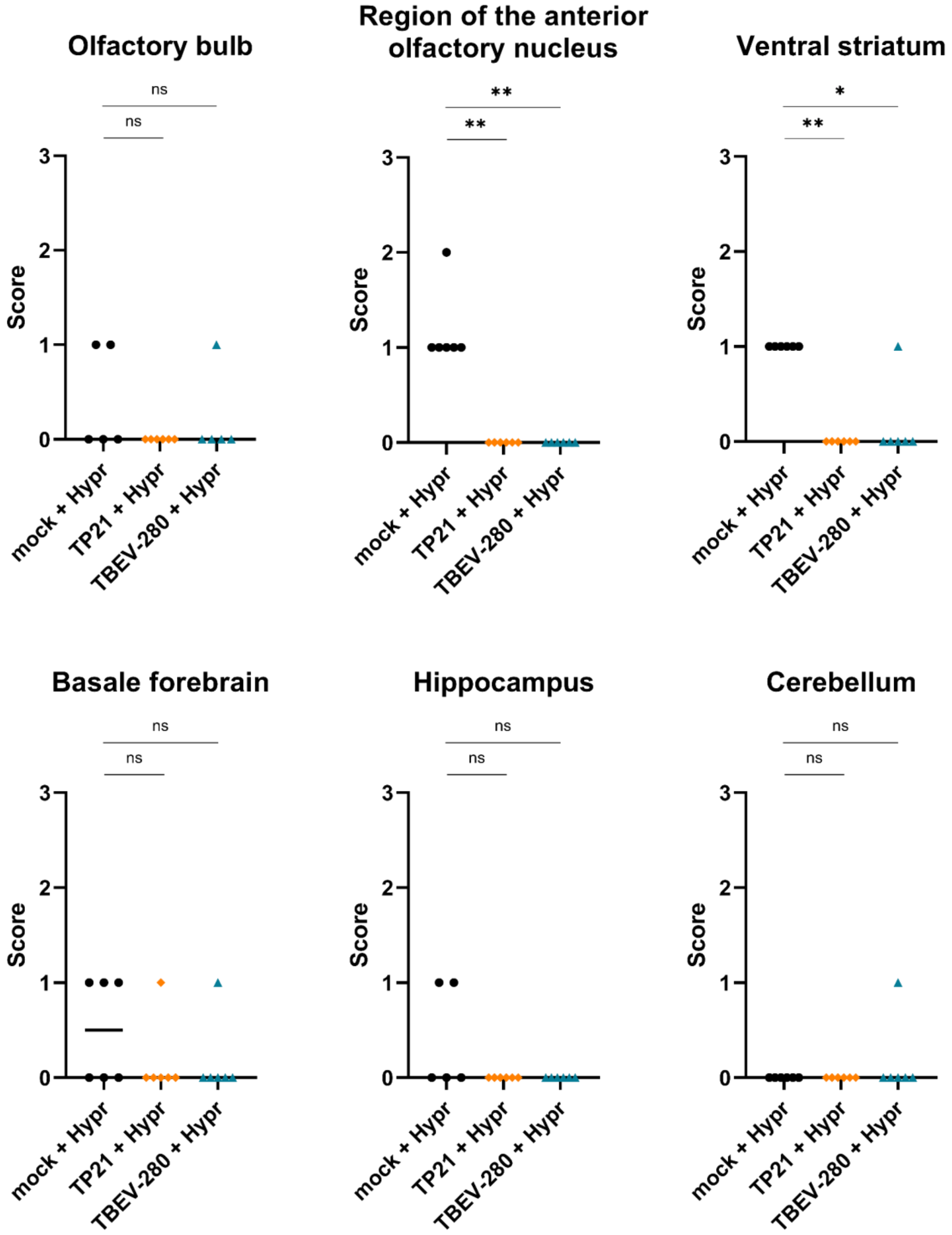

Supplement: Supplementary file 1 [file vaccines-09-00196-s001.pdf]
